# Arabidopsis paralogous genes *RPL23aA* and *RPL23aB* encode functionally equivalent proteins

**DOI:** 10.1186/s12870-020-02672-1

**Published:** 2020-10-08

**Authors:** Wei Xiong, Xiangze Chen, Chengxin Zhu, Jiancong Zhang, Ting Lan, Lin Liu, Beixin Mo, Xuemei Chen

**Affiliations:** 1 Guangdong Provincial Key Laboratory for Plant Epigenetics, Longhua Bioindustry and Innovation Research Institute, College of Life Sciences and Oceanography, Shenzhen University, Shenzhen, 518060 Guangdong China; 2 Key Laboratory of Optoelectronic Devices and Systems of Ministry of Education and Guangdong Province, College of Optoelectronic Engineering, Shenzhen University, Shenzhen, Guangdong 518060 China; 3 Department of Botany and Plant Sciences, Institute of Integrative Genome Biology, University of California, Riverside, CA 92521 USA

**Keywords:** Ribosome, Ribosomal protein, RPL23a, Gene dosage, Paralogue, Functional specialization

## Abstract

**Background:**

In plants, each ribosomal protein (RP) is encoded by a small gene family but it is largely unknown whether the family members are functionally diversified. There are two *RPL23a* paralogous genes (*RPL23aA* and *RPL23aB*) encoding cytoplasmic ribosomal proteins in *Arabidopsis thaliana*. Knock-down of *RPL23aA* using RNAi impeded growth and led to morphological abnormalities, whereas knock-out of *RPL23aB* had no observable phenotype, thus these two RPL23a paralogous proteins have been used as examples of ribosomal protein paralogues with functional divergence in many published papers.

**Results:**

In this study, we characterized T-DNA insertion mutants of *RPL23aA* and *RPL23aB*. A rare non-allelic non-complementation phenomenon was found in the F1 progeny of the *rpl23aa* X *rpl23ab* cross, which revealed a dosage effect of these two genes. Both *RPL23aA* and *RPL23aB* were found to be expressed almost in all examined tissues as revealed by GUS reporter analysis. Expression of *RPL23aB* driven by the *RPL23aA* promoter can rescue the phenotype of *rpl23aa*, indicating these two proteins are actually equivalent in function. Interestingly, based on the publicly available RNA-seq data, we found that these two *RPL23a* paralogues were expressed in a concerted manner and the expression level of *RPL23aA* was much higher than that of *RPL23aB* at different developmental stages and in different tissues.

**Conclusions:**

Our findings suggest that the two RPL23a paralogous proteins are functionally equivalent but the two genes are not. *RPL23aA* plays a predominant role due to its higher expression levels. *RPL23aB* plays a lesser role due to its lower expression. The presence of paralogous genes for the RPL23a protein in plants might be necessary to maintain its adequate dosage.

## Background

Ribosomes are responsible for protein synthesis in all living cells. A single ribosome is a ribonucleoprotein complex formed from a large and a small subunit. In plants, the large ribosomal subunit is composed of 28S, 5.8S and 5S rRNAs together with 48 RPL (Ribosomal Protein of Large subunit) proteins, whereas the small subunit is composed of 18S rRNA and 33 RPS (Ribosomal Protein of Small subunit) proteins [1, 2]. In *E. coli*, genes encoding RPs are arranged in about 20 operons, with approximately half of the genes mapping to a single locus [3, 4]. In mammals, although there are about 2000 sequences which may encode RPs, most of them are predicted to be pseudogenes, and most functional RPs are encoded by a single copy [5]. In yeast *Saccharomyces cerevisiae*, about 75% of the RPs are encoded by gene families with more than one member [6]. Although substantially functional redundancy was found between paralogous RP genes in yeast, some paralogous RP genes were reported to have non-redundant functions [6–8].

Plants have even more gene members encoding a single RP than yeast [9]. In *Arabidopsis thaliana*, RP paralogues share 65 to 100% amino acid sequence identity [9]. Assessment of cognate EST (expressed sequence tag) numbers of RP genes suggested that RP gene family members were differentially expressed in Arabidopsis [9]. Microarray data also revealed that transcripts of RP genes within the same family were accumulated at different levels in Arabidopsis [10]. Under various stimuli, while the transcript levels for most RP genes remain unchanged, some RP genes show altered expression levels [10]. Many studies have investigated the phenotypic consequence of absent or reduced expression of a single RP paralogue in Arabidopsis. Disruptions in any one of the RP protein genes, *RPL3A*, *RPL8A*, *RPL19A*, *RPL23C*, *RPL40B*, and *RPS11A* is embryo lethal [11]. Less severe phenotypes were reported for mutations in several other RPs [11]. Morphological changes of the first two true leaves from the spatulate wild type shape to a pointed shape were found in mutants of some RP genes, including *RPL5A*, *RPL5B*, *RPL9C*, *RPL10aB*, *RPL24B*, *RPL28A*, *RPS13B*, and *RPS18A* [12–16]*.* Despite these studies on RPs, it remains unknown why RPs are encoded by paralogues in plants or whether RP paralogues have specialized functions.

In Arabidopsis, the *RPL23a* family consists of two members (*RPL23aA* and *RPL23aB*) that encode proteins with 95% amino acid identity. Both *RPL23aA* and *RPL23aB* genes are transcribed and translated, and protein products of either paralogue can be incorporated into the cytoplasmic ribosome [17, 18]. Knock-down of the *RPL23aA* gene through RNAi results in severe developmental defects, whereas knock-down, or even knock-out, of *RPL23aB* has no obviously phenotypic consequences [19], which could be the basis for the argument that *RPL23aA* and *RPL23aB* had specialized functions [11, 19–21].

With the general question of why plant RPs are encoded by paralogous genes in mind, we sought to study the functional relationship between *RPL23aA* and *RPL23aB*. With T-DNA insertion mutants in *RPL23aA* and *RPL23aB*, we found a rare non-allelic non-complementation phenomenon, indicating that these two *RPL23a* genes are dosage dependent genes. We showed that expression of *RPL23aB* driven by the *RPL23aA* promoter can rescue the phenotype of *rpl23aa*, demonstrating that RPL23aA and RPL23aB proteins are functionally equivalent. Furthermore, interrogation of RNA-seq data from several developmental stages and in different organs showed that although the level of *RPL23aA* transcripts was much higher than that of *RPL23aB*, the fluctuations in expression of the two genes were well matched, suggesting that these two genes were coordinately regulated. These results revealed that duplicated *RPL23a* genes contribute to ribosome dosage necessary for plant growth and development. Our results do not contradict prior studies showing that *RPL23aA* plays a dominant role in plant growth and development, but reveal that the *RPL23aA* dominance resides in its higher expression level rather than functional specialization of the protein.

## Methods

### Plant material and growth conditions

*Arabidopsis thaliana* wild type Columbia-0 (Col-0) and the T-DNA insertion lines, *SALK_005448* (named here *rpl23aa*) and *SAIL_597_B08* (named here as *rpl23ab*), were obtained from the Arabidopsis Biological Resource Center (ABRC). Seeds were first treated for 2 min in 75% ethanol, then treated for 6 min in commercial bleach and rinsed at least 3 times with sterile distilled water. Solid medium consisted of 2.2 g/L Murashige and Skoog basal salt mixture (Phyto Tech Labs), 10 g/L sucrose, and 8 g/L agar. pH was adjusted to 5.6 with KOH before autoclaving. When required, BASTA (GOLDBIO) was added at a final concentration of 125 μg/L. Seeds were sown in a water suspension, using a 1.5 mL pipette tip, in 150 mm Petri dishes filled with 120 ml of solid culture medium, at a density of 150 regularly spaced seeds per plate. Once inoculated, the Petri dishes were sealed with Micropore Scotch 3 M surgical tape, which prevented contamination but allowed gaseous exchange, and placed in 4 °C for 24 h. Growth was allowed to proceed at 22 °C in Percival tissue culture chambers under long day conditions (16 h light and 8 h dark). 10-day seedlings were then transplanted to pots containing a 1:2:2 mixture of perlite, vermiculite and soil at 22 °C under long day conditions from a combination of incandescent and fluorescent lamps (10,000 lx). Plants were watered twice a week with nutrient solution.

### RNA isolation and RT-PCR

50 mg seedlings from 14-day-old Col-0, *rpl23aa*, and *rpl23ab* were harvested and immediately frozen in liquid nitrogen. RNA was extracted using RNAiso Plus (TAKARA BIO INC). In the elution step, RNA was resuspended in DEPC-treated water. cDNA was obtained by reverse transcription of 1 μg of RNA with the PrimeScriptTMRT reagent Kit with gDNA Eraser (TAKARA BIO INC).

### Plasmid construction and generation of transgenic plants

In order to construct the pRPL23aA::RPL23aA and pRPL23aB::RPL23aB plasmids, a 3001 bp DNA fragment (including the promoter region) of *RPL23aA* (AT2G39460) and a 2016 bp DNA fragment (including the promoter region) of *RPL23aB* (AT3G55280) were amplified from Col-0 genomic DNA using Phusion polymerase (Thermo Scientific). The primers used are shown in Table S1 (Additional file 12). The amplified DNA sequences were cloned in pEG301 [22] to result in pRPL23aA::RPL23aA and pRPL23aB::RPL23aB*.* The plasmids were used to transform *rpl23aa*. For pRPL23aA::RPL23aB construction, the promoter region (about 1.5 kb) of *RPL23aA* plus the coding region of *RPL23aB* were synthesized by a commercial company (GENEWIZ SuZhou), then the synthesized DNA fragment was sequenced and was cloned in pEG301. The promoter regions of *RPL23aA* (about 1.5 kb) and *RPL23aB* (about 1.5 kb) were cloned into pMDC162 [22] to generate the plasmids pRPL23aA::GUS and pRPL23aB::GUS, which were then used to transform Col-0 plants. Floral dip transformation was performed as described by Clough and Bent [23]. T1 transgenic plants were screened on solid 1/2 Murashige & Skoog (MS) medium with 25 mg/L Hygromycin B or 0.002% BASTA and verified by PCR. GUS staining was carried out with plants in the T2 generation.

### GUS staining assay

8-days-old seedlings and 36-days-old inflorescences, immature and mature flowers, immature and mature siliques of Col-0, *pRPL23aA:GUS* and *pRPL23aB:GUS* were subjected to histochemical GUS staining according to the standard protocol [24].

### Transcripts profiling

RNA-seq data was obtained from a public website (http://travadb.org/browse/DeSeq/), and the average value of normalized absolute read counts from two biological replicates was extracted. We also downloaded the original RNA-seq data of *A. thaliana* different organs and developmental stages from NCBI Sequence Read Archive (project ID PRJNA314076 for samples except meristem and project ID PRJNA268115 for the meristem samples). The RPKM (Reads Per Kilobase per Million mapped reads) value of *RPL23aA* (AT2G39460), *RPL23aB* (*AT3G55280*), and *ACT2* (AT3G18780) were calculated. Our calculated RPKM value is consistent with the value of normalized absolute read counts obtained from the public website (http://travadb.org/browse/DeSeq/).

### Polysome profiling

Polysome profiling was performed as described by Mustroph et al. [25]. Briefly, 2 g of 14-day-old seedlings were collected and ground to a fine powder using sufficient liquid nitrogen, and the powder was resuspended in 8 mL of ice-cold polysome extraction buffer by gentle shaking. The lysate was incubated on ice for 10 min and centrifuged at 4 °C, 16, 000 x g for 15 min. The supernatant was filtered through Miracloth and centrifuged at 4 °C, 16, 000 x g for another 15 min. The supernatant was gently transferred to the top of a sucrose cushion and then centrifuged at 4 °C, 50,000 r.p.m. for 3 h to obtain the polysome pellet. The pellet was resuspended in ice-cold resuspension buffer and loaded onto a 4.5 mL sucrose gradient (20–60% w/v) for fractionation of polysomes by ultracentrifugation, after which the sucrose gradient was pumped through a UV detector and absorbance at 254 nm was recorded.

## Results

### Characterization of *rpl23aa* and *rpl23ab* mutants

**Fig. 1:**
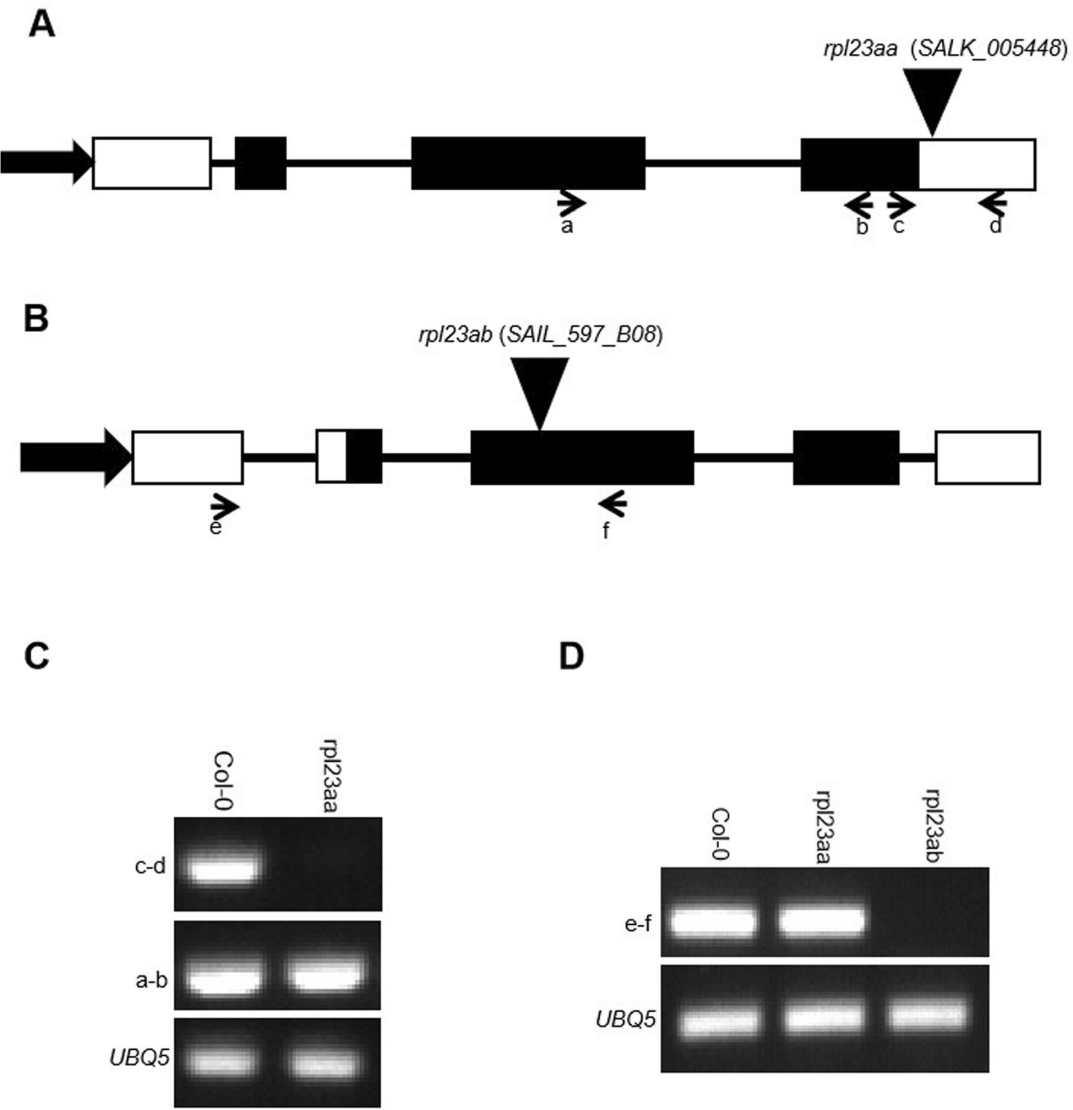
Characterization of *rpl23aa* and *rpl23ab* mutants. **a**, **b** Structure of the *RPL23aA* and *RPL23aB* paralogous genes, with the positions of the T-DNA insertions in *rpl23aa* and *rpl23ab* mutants indicated by black triangles. Black boxes and lines between black boxes indicate exons and introns, respectively. White boxes correspond to the 5′ and 3′ untranslated regions. Long arrows indicate promoters. Short arrows represent primers used in RT-PCR in **c** and **d**. **c**, **d** Semi-quantitative RT-PCR analysis of *RPL23aA* and *RPL23aB* transcripts in the corresponding mutant background. The size of PCR products: a-b 200 bp, c-d 200 bp, and e-f 200 bp. The full-length gel of **c** is presented in Supplementary Figure S8 (Additional file 8), and the full-length gel of **d** is presented in Supplementary Figure S9 (Additional file 9)

The Arabidopsis genome contains two *RPL23a* paralogous genes *RPL23aA* (At2g39460) and *RPL23aB* (At3g55280), which encode proteins with 95% amino acids identity (see Additional file 1). We acquired T-DNA insertion lines of *RPL23aA* and *RPL23aB*, namely *SALK_005448 and SAIL-597-B08*, respectively (hereafter referred to as *rpl23aa* and *rpl23ab*). PCR-genotyping confirmed that both *rpl23aa* and *rpl23ab* are homozygous T-DNA insertion alleles (see Additional file 2). Sequencing results revealed that *rpl23aa* contains a T-DNA insertion in the 3′ UTR region, 10 bp downstream of the stop codon of the *RPL23aA* gene (Fig. 1a), while *rpl23ab* contains a T-DNA insertion in the second exon of *RPL23aB* (Fig. 1b). A semi-quantitative RT-PCR assay was used to detect transcripts from *RPL23aA* and *RPL23aB* in these T-DNA lines. As shown in Fig. 1c, the 3′ region around the stop codon of the *RPL23aA* mRNA was disrupted in the mutant. Because the majority of the *RPL23aA* mRNA from the T-DNA line was intact, we suspect that *SALK_005448* is a hypomorphic allele. *rpl23ab* is likely a null mutant, because no *RPL23aB* mRNA was detected (Fig. 1d). Absence of dosage compensation by *RPL23aA* in Arabidopsis was reported following loss of *RPL23aB* [26]. As shown in Fig. 1d, there is also no dosage compensation by *RPL23aB* in the *rpl23aa* mutant.

**Fig. 2:**
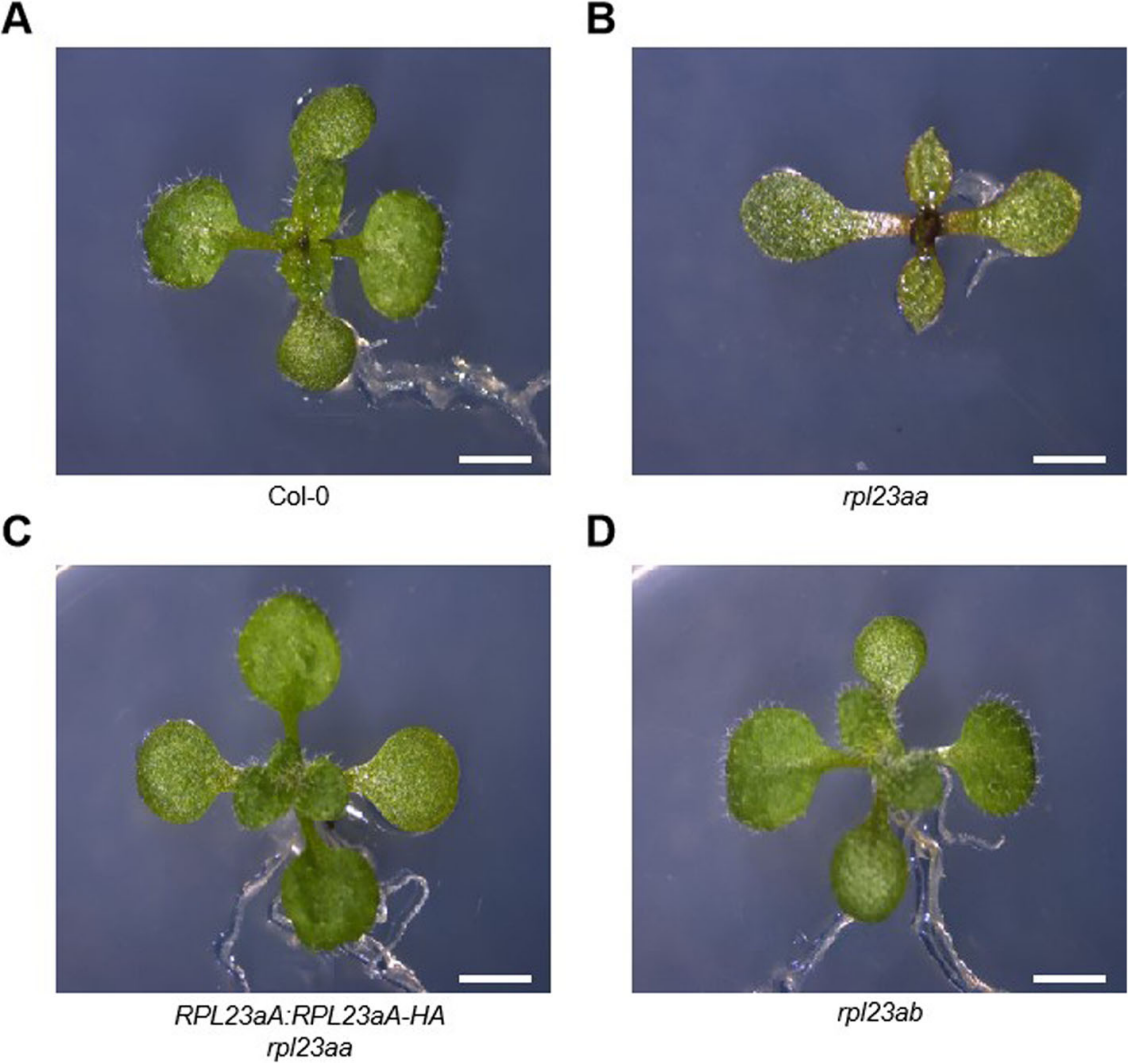
Plant phenotypes. 14-day-old plants of (**a**) Col-0, (**b**) *rpl23aa,* (**c**) p*RPL23aA*::*RPL23aA-HA*/*rpl23aa,* (**d**) *rpl23ab. rpl23aa* exhibits pleiotropic defects, including pointed leaves, retarded root growth, and reduced plant size; *pRPL23aA*::*RPL23aA-HA* fully rescued the morphological defects of *rpl23aa; rpl23ab* had no observable phenotype. Size bar, 2 mm

The *rpl23aa* mutant exhibits pleiotropic defects, including pointed leaves, retarded root growth, and reduced plant size (Fig. 2b). These phenotypes are similar to those of a previously reported RNAi line [19]. An incompletely penetrant tricotyledon phenotype (less than 5% of the total population) was observed in *rpl23aa* mutant plants (see Additional file 3). However, we didn’t observe appreciable defects in terms of growth rate, morphology, or flowering in the *rpl23ab* mutant (Fig. 2d), which is consistent with published work [26]. We amplified genomic DNA encompassing the promoter plus the coding region of *RPL23aA* from wild-type plants and fused it to the sequence encoding the HA epitope tag. When this transgene was introduced into *rpl23aa*, the developmental defects were fully rescued (Fig. 2c), suggesting that dysfunction of *RPL23aA* was responsible for the developmental defects in *rpl23aa*.

### *RPL23aA* and *RPL23aB* are dosage-dependent genes

**Fig. 3:**
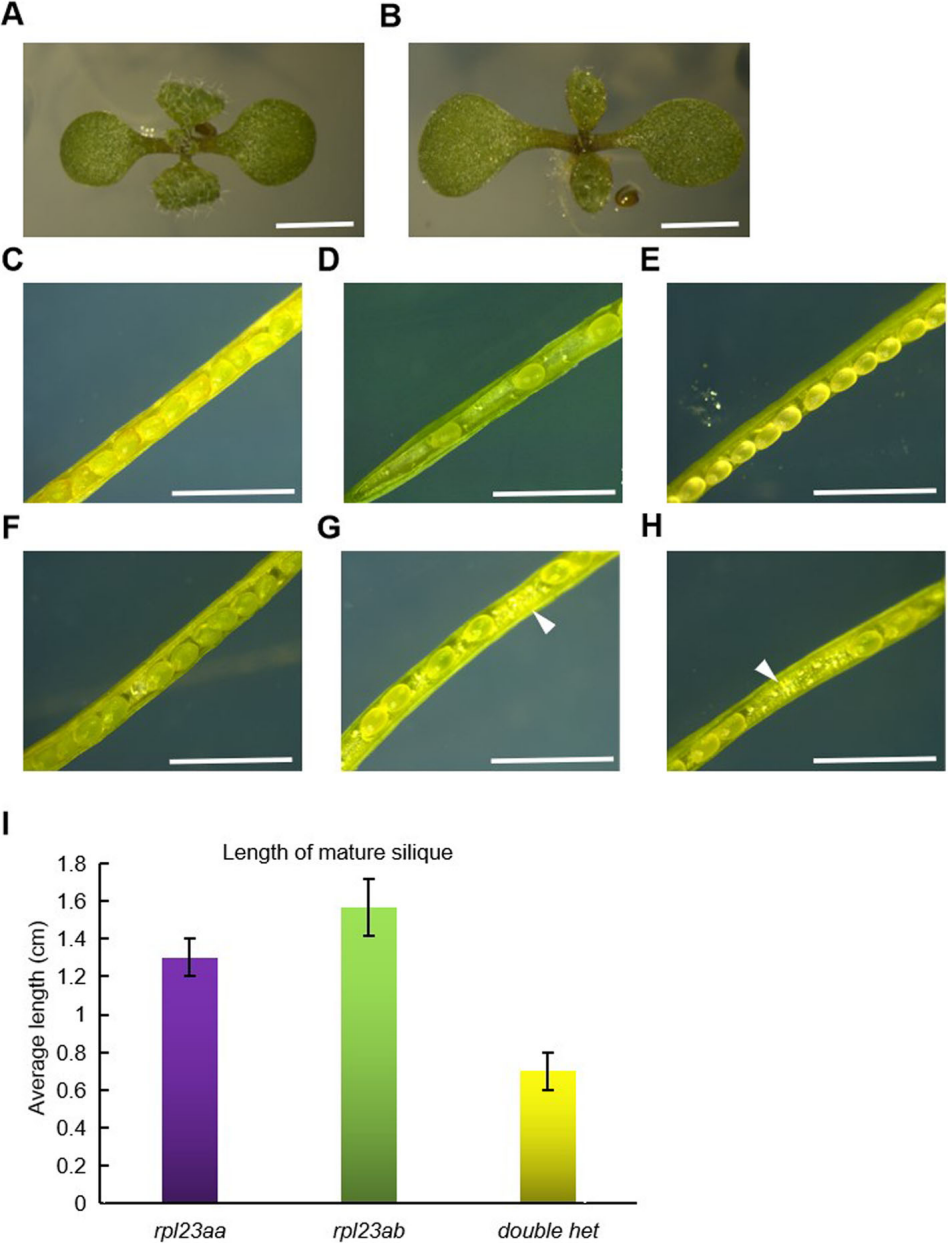
The non-allelic non-complementation phenomenon between *rpl23aa* and *rpl23ab*. 9-day-old plants of (**a**) *rpl23aa* × Col-0 (F1 generation)*,* (**b**) *rpl23aa* × *rpl23ab* (F1 generation). Dissected mature siliques from (**c**) Col-0, (**d**) *rpl23aa*, (**e**) *rpl23ab*, (**f**) *rpl23aa* × Col (F1 generation), (**g**) *rpl23aa* × *rpl23ab* (F1 generation), (**h**) *rpl23ab* × *rpl23aa* (F1 generation). (**i**) The length of mature silique from *rpl23ab, rpl23aa,* and the double heterozygote (double het). Arrowheads indicate aborted embryos. Size bar, 5 mm

In order to study the genetic interaction between *RPL23aA* and *RPL23aB*, we crossed *rpl23aa* with *rpl23ab*. To our surprise, the doubly heterozygous plants (*RPL23aA*/*rpl23aa*; *RPL23aB*/*rpl23ab*) in the F1 progeny all have pointed first true leaves (Fig. 3b). Siliques of the doubly heterozygous plants are much shorter than siliques of *rpl23aa* or *rpl23ab* (Fig. 3i). We dissected siliques from *RPL23aA*/*rpl23aa*; *RPL23aB*/*rpl23ab* plants and found many aborted ovules (Fig. 3g and h). An F2 population was generated by selfing the above F1 plants. We genotyped 144 F2 plants but did not find double homozygous (*rpl23aa* /*rpl23aa*; *rpl23ab* /*rpl23ab*) plants. In fact, we did not even detect any genotypes with a single functional allele from either gene (*RPL23aA*/*rpl23aa*; *rpl23ab* /*rpl23ab or rpl23aa* /*rpl23aa*; *RPL23aB*/*rpl23ab*) (Table 1), although these genotypes are collectively expected to appear in 31.25% (5 out of 16) of the F2 plants. We suspected that this non-allelic non-complementation phenomenon between *rpl23aa* and *rpl23ab* is probably due to gene dosage effects.

**Table 1:** Genetic interactions between *rpl23aa* and *rpl23ab*

Genotype	First leaf phenotype	
	Pointed	Normal
*RPL23aA/RPL23aA RPL23aB/RPL23aB*	0	15
*RPL23aA/RPL23aA RPL23aB/rpl23ab*	0	37
*RPL23aA/RPL23aA rpl23ab/rpl23ab*	0	19
*RPL23aA/rpl23aa RPL23aB/RPL23aB*	0	26
*RPL23aA/rpl23aa RPL23aB/rpl23ab*	38	0
*RPL23aA/rpl23aa rpl23ab/rpl23ab*	0	0
*rpl23aa/rpl23aa RPL23aB/RPL23aB*	9	0
*rpl23aa/rpl23aa RPL23aB/rpl23ab*	0	0
*rpl23aa/rpl23aa rpl23ab/rpl23ab*	0	0

*rpl23aa* and *rpl23ab* were crossed and the F_2_ plants were subjected to genotyping at the *RPL23aA* and *RPL23aB* loci. Leaf phenotype of the plants was classified into pointed or normal. Primers for genotyping are listed in Table S1 (Additional file 12)

### *RPL23aA* and *RPL23aB* genes have similar expression patterns

**Fig. 4:**
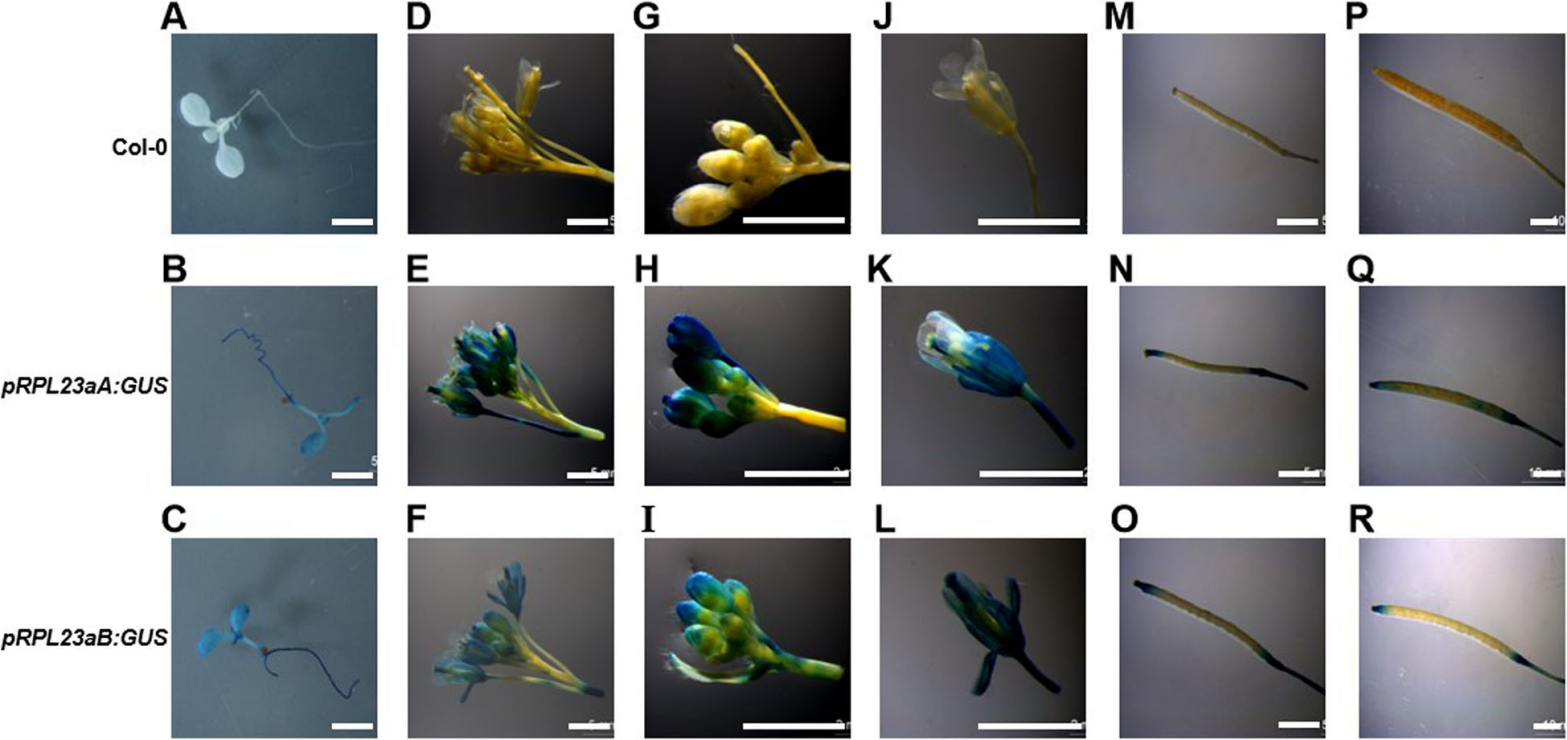
Promoter-GUS reporter analysis of *RPL23aA* and *RPL23aB.* **a**, **b**, **c** Seedling. **d**, **e**, **f** Inflorescences. **g**, **h**, **i** Immature and **j**, **k**, **l** mature flowers. **m**, **n**, **o** Immature and **p**, **q**, **r** mature siliques. Pictures were taken at 8 days **a**-**c** and 36 days **d**-**r**. Size bar, 5 mm

In order to investigate the expression patterns of *RPL23aA* and *RPL23aB* genes, we fused the promoter regions of *RPL23aA* and *RPL23aB* genes to the GUS reporter and generated transgenic plants in the Col-0 background. GUS staining of 14 *pRPL23aA:GUS* and 5 *pRPL23aB:GUS* independent transgenic lines uncovered a ubiquitous expression pattern for both genes with particularly intense GUS staining in young and actively proliferating tissues, such as developing leaves, floral buds and root apices (Fig. 4). Similar expression patterns of *RPL23aA* and *RPL23aB* support our hypothesis that the non-allelic non-complementation phenomenon between these two genes is the consequence of overlap in expression (and function) of *RPL23aA* and *RPL23aB* in the same cells.

### RPL23aA and RPL23aB proteins are functionally equivalent

It has been reported that some paralogous ribosomal proteins have evolved specialized functions in yeast [6]. As mentioned above, dysfunction of *RPL23aA* results in severe developmental defects, whereas knock-out of *RPL23aB* has no phenotypic consequences in Arabidopsis ([19, 26] and this study). It’s natural to assume that these two paralogous ribosomal proteins have undergone functional specialization.

**Fig. 5:**
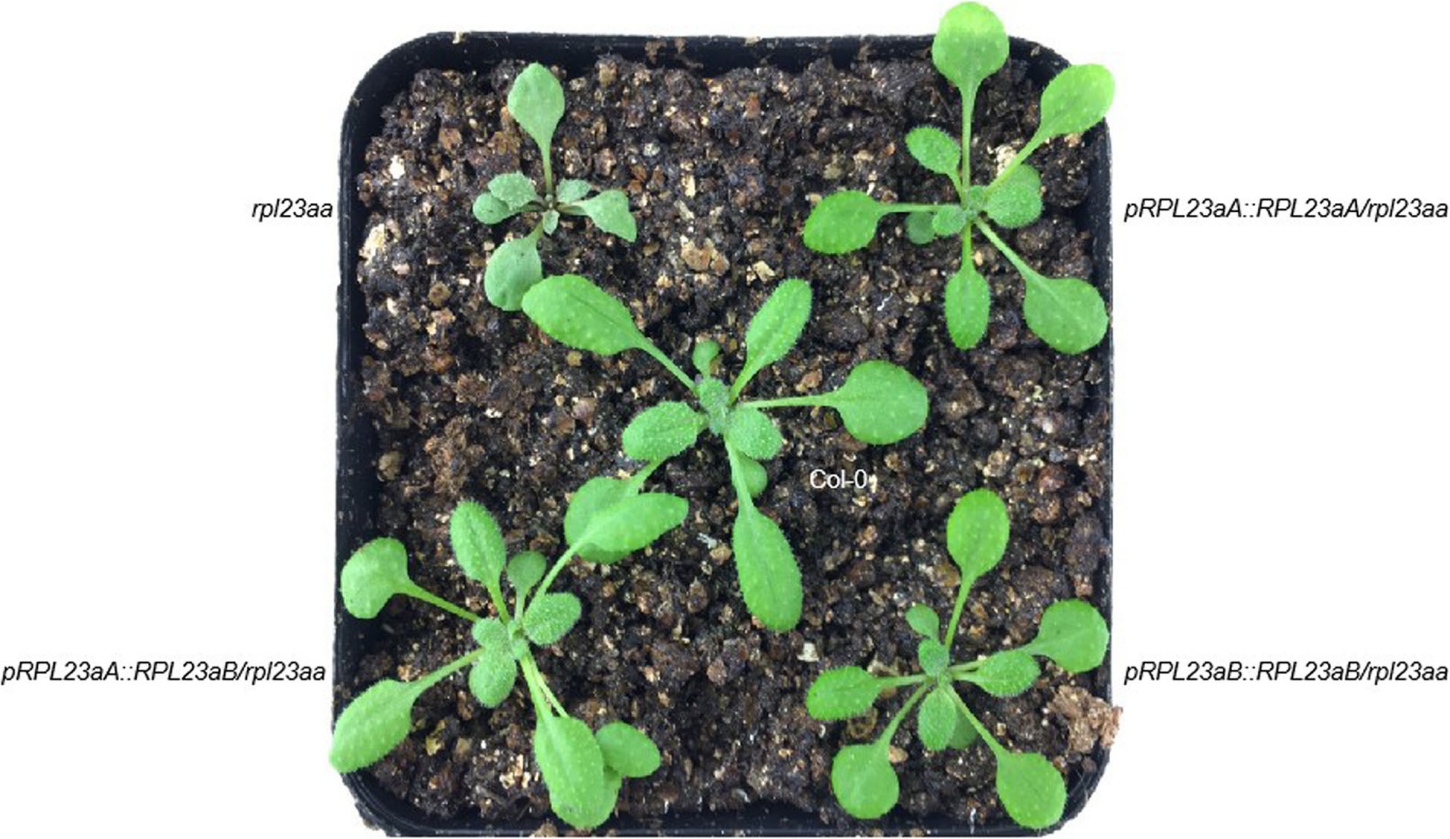
Phenotypes of representative 17-day-old plants. Upper left:*rpl23aa;* upper right**:** *pRPL23aA::RPL23aA/rpl23aa;* lower left**:** *pRPL23aA::RPL23aB/rpl23aa*; lower right: *pRPL23aB::RPL23aB/rpl23aa*; central: Col-0

We designed gene complementation experiments to explore whether RPL23aA and RPL23aB have distinct functions. If RPL23aA and RPL23aB have specialized functions, RPL23aB is not expected to complement the *rpl23aa* mutation. We fused the promoter regions of *RPL23aA* to the coding region of *RPL23aB*. The *pRPL23aA:RPL23aB* transgene was introduced into *rpl23aa* plants, and 21 independent *pRPL23aA:RPL23aB* transgene lines were obtained, among which 15 lines rescued the phenotype of *rpl23aa* (Fig. 5 and Additional file 4), indicating that RPL23aA and RPL23aB have equivalent function. The *pRPL23aB:RPL23aB* transgene was also introduced into *rpl23aa* plants, and 8 out of 15 independent, homozygous transgenic lines exhibited near wild type morphology (Fig. 5 and Additional file 4). However, a portion (about 2%) of the transgenic plants of each line exhibited the tricotyledon phenotype (see Additional file 5). Thus, the *pRPL23aB:RPL23aB* transgene can largely but not fully rescue the phenotype of *rpl23aa*.

### *RPL23aA* and *RPL23aB* genes are transcribed in a concerted manner with higher expression levels of *RPL23aA* than *RPL23aB*

**Fig. 6:**
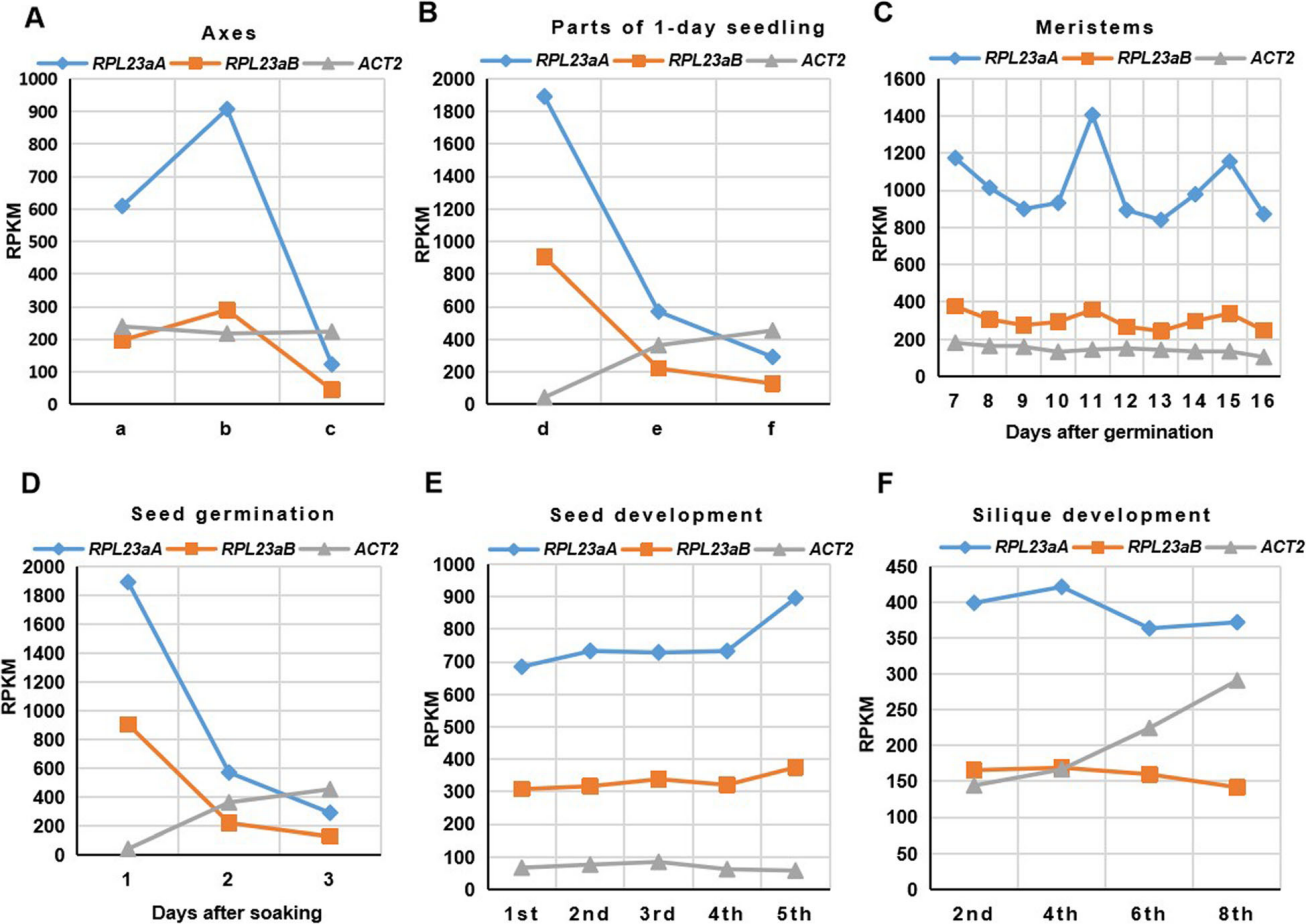
Transcript profiles of *RPL23aA* and *RPL23aB* at different developmental stages and in different organs. Y axis: the average RPKM (Reads Per Kilobase per Million mapped reads) value of two biological replicates. **a** Different parts of axes. a, peduncles; b, inflorescence axis; c, the first elongated internode; **b** Parts of 1-day-old seedling. d, hypocotyl; e, cotyledons; f, apical meristem with adjacent tissues. **c** Meristems after germination. **d** Seed germination after soaking. **e** Seed development. The X-axis represents the siliques from which ovules were taken at the moment when the first silique was 1 cm long. **f** Silique development. The X-axis represents siliques (seeds not removed) sampled at the moment when the first silique was 1 cm long

Since the above results indicated that RPL23aA and RPL23aB proteins have equivalent function, we suspected that the difference in phenotype between *rpl23aa* and *rpl23ab* is due to the difference in the expression levels of these two genes. The expression of *RPL23aA* may be much higher than *RPL23aB*, so the impacts on ribosomes by the *rpl23aa* mutation are higher than the *rpl23ab* mutation thus leading to much severe morphological defects*.* We compared the transcript levels of *RPL23aA* and *RPL23aB* at different developmental stages and in different organs by analyzing published RNA-seq data [27]. As shown in Fig. 6 and Figure S6(Additional file 6), transcript levels of *RPL23aA* are much higher than those of *RPL23aB* at all developmental stages and in all the examined tissues. Strikingly, the spatial and temporal patterns of expression of these two paralogous genes are well matched, suggesting that they are similarly regulated at differently developmental stages in all examined tissues. *ACT2*, which is a house keeping gene, was included as a control. Transcript levels of *RPL23aB* are higher than *ACT2* in some organs, and total amount of *PRL23a* transcripts is much higher than *ACT2* in most examined organs (Fig. 6c, e), indicating that RPs are in great demand for plant development.

### The polysome/monosome ratio is elevated in the *rpl23a* mutants

In order to evaluate the effects of the *rpl23aa* and *rpl23ab* mutations on polysomes, we fractionated total ribosomes by ultracentrifugation through sucrose density gradients. The polysome profiles from plants of various genotypes are shown in Figure S7 (Additional file 7). To our surprise, the ratio between polysomes and the monosome is obviously increased in *rpl23aa* and slightly increased in *rpl23ab* compared to wildtype. The increase in the polysome/monosome ratio in *rpl23aa* was largely rescued by both *pRPL23aA:RPL23aA* and *pRPL23aA:RPL23aB* transgenes, whereas the polysome/monosome ratio in *pRPL23aB:RPL23aB/rpl23aa* transgenic plants is higher than wildtpye and lower than *rpl23aa*. The changes in ribosomal profile of *rpl23aa* and *rpl23ab* indicate that the overall translation state is altered. The higher polysome levels could reflect higher rates of translation or defects in translation, such as slower elongation. While the molecular basis of the higher polysome levels is unknown, the stronger effect of the *rpl23aa* mutation is consistent with the dominant role of *RPL23aA* over *RPL23aB* as suggested by expression levels and mutant phenotypes.

## Discussion

Some of the paralogous RPs are identical in amino acid sequences such as RPL36aA and RPL36aB, but many of the paralogues display sequence variations and are differentially expressed during development. The presence of multiple gene members for each RP in plants might be necessary to maintain adequate RP doses or to maintain some degree of ribosome heterogeneity and functional specialization.

In this study, we characterized the *RPL23a* gene family containing two highly homologous family members. The hypomorphic T-DNA insertion allele of *RPL23aA* exhibits pleiotropic defects. However, knock-out of *RPL23aB* has no appreciable phenotypic impacts. We crossed mutants of *RPL23aA* and *RPL23aB* and found a non-allelic non-complementation phenomenon in their F1 progeny. This phenomenon is also found in other RP coding gene families such as *RPL5* [28]*, RPL36a* [29], and *RPS6* [30]. However, mutations in the paralogues within *RPL5, RPL36a*, and *RPS6* families caused almost the same phenotype, indicating that the paralogues are functionally equivalent. In the case of the *RPL23a* family, phenotypes of the single mutants suggest unequal functions of the two paralogues. The non-allelic non-complementation phenomenon may be due to a dosage problem - reduced dosage at one of the paralogues still supports the wild phenotype but simultaneous reduction of dosage at both paralogues could not sustain the wild phenotype. For the dosage effect hypothesis to be true, there must be at least some overlap in the expression of the gene family members. Indeed, promoter-GUS experiments demonstrated that both *RPL23aA* and *RPL23aB* were ubiquitously expressed.

Phenotypical differences between members of an RP within a family might result from diversification of protein function or variation in levels and patterns of expression. We demonstrated that RPL23aA and RPL23aB proteins had equal function, as expression of *RPL23aB* driven by the *RPL23aA* promoter could rescue the phenotype of the *rpl23aa* mutant. We found that the expression level of *RPL23aA* was much higher than that of *RPL23aB* according to the publicly available RNA-seq data. Thus, the difference in expression levels might be the reason why disruption of *RPL23aA* and *RPL23aB* had different consequences. It is interesting that despite the difference in expression levels, the temporal and spatial patterns of expression of the two paralogous genes were nearly identical. These results suggested that *RPL23aA* and *RPL23aB* genes are transcribed in a coordinated manner. Posttranscriptional and translational regulation may also play a role in *RPL23aA* and *RPL23aB* expression [31]. Subcellular localization specialization could be another factor that causes differences in functional effects between paralogous RPs [32]. Previous studies revealed that both of RPL23aA and RPL23aB are targeted to the nucleolus with RPL23aA targeting being a bit more efficient than RPL23aB [10, 19]. Targeting of RP to the nucleolus is an essential step in eukaryotic ribosome biogenesis [33, 34], so the efficiency of RPL23aA assembly into ribosomes may be higher than that of RPL23aB. Although posttranscriptional differences between RPL23aA and RPL23aB may exist, the fact that expression of *RPL23aB* with the *RPL23aA* promoter rescues the *rpl23aa* phenotypes indicates that differences in expression level underlie the different functional contributions of the paralogues as exemplified by the single mutant phenotypes.

There are at least four possible consequences of a RP disruption: (1) ribosome insufficiency, (2) non-functional ribosomes, (3) partially dysfuntional ribosomes, and (4) loss of the extraribosomal funtion of the RP [35]. Polysome profiling results revealed that the polysome/monosome ratio is elevated in the *rpl23a* mutants, which suggested global translational alteration. The exact nature of the alteration remains unknown and will be investigated in the future.

## Conclusions

Ribosomal protien RPL23a paralogues (RPL23aA and RPL23aB) have been used as examples of paralogues with functional divergence in many published papers. In this study, our findings provided four convincing evidences demonstrating duplicated *RPL23a* genes actually have redundant function (without functional specialization), thus are necessary to provide a threshold dose: 1) The non-allelic non-complementation phenomenon between *rpl23aa* and *rpl23ab* suggests *RPL23aA* and *RPL23aB* are dosage dependent genes; 2) *RPL23aA* and *RPL23aB* genes are expressed in the same tissues; 3) RPL23aB could rescue the phenotype of *rpl23aa*, demonstrating RPL23aA and RPL23aB protein have equal function; 4) *RPL23aA* and *RPL23aB* genes are transcribed in a concerted manner with higher expression levels of *RPL23aA* than *RPL23aB*. Our findings suggest that the two paralogous RPL23a proteins have equivalent function and the presence of multiple genes for individual RPs in plants might be necessary to maintain adequate ribosome dosage at least for some ribosomal protein families.

## Supplementary information

**Additional file 1: Figure S1**. Amino acid sequence alignment between RPL23aA and RPL23aB.

**Additional file 2: Figure S2**. Genotyping of *rpl23aa* and *rpl23ab.*

**Additional file 3: Figure S3**. Images of wild type and *rpl23aa* plants.

**Additional file 4: Figure S4**. Lengths of mature siliques and numbers of ovules in mature siliques.

**Additional file 5: Figure S5**. Images of *pRPL23aB:RPL23aB rpl23aa* plants.

**Additional file 6: Figure S6**. Transcript profiles of *RPL23aA* and *RPL23aB* in different organs.

**Additional file 7: Figure S7**. Polysome profiles of Col-0 (black), *rpl23aa* (red), *rpl23ab* (green), *pRPL23aA:RPL23aA/rpl23aa* (yellow), *pRPL23aA:RPL23aB/rpl23aa* (blue), and *pRPL23aB:RPL23aB/rpl23aa* (purple).

**Additional file 8: Figure S8**. Full-length gel of Fig. 1c.

**Additional file 9: Figure S9**. Full-length gel of Fig. 1d.

**Additional file 10: Figure S10**. Full-length gel of Figure S2C.

**Additional file 11: Figure S11**. Full-length gel of Figure S2D.

**Additional file 12: Table S1**. Primers used in this work.

## Data Availability

The original RNA-seq data of *A. thaliana* different organs and developmental stages were downloaded from NCBI Sequence Read Archive (project ID PRJNA314076 for samples except meristem and project ID PRJNA268115 for the meristem samples).

## Abbreviations

RP: ribosomal protein
RPL: ribosomal protein of large subunit
RPS: ribosomal protein of small subunit
RPKM: Reads Per Kilobase per Million mapped reads
