# Supplementary material for: Arabidopsis paralogous genes RPL23aA and RPL23aB encode functionally equivalent proteins
Source: BMC Plant Biol. 2020 Oct 8;20:463. doi: 10.1186/s12870-020-02672-1 (PMC7545930; doi:10.1186/s12870-020-02672-1)
Supplement: Supplementary file 1 — Additional file 1: Figure S1. Amino acid sequence alignment between RPL23aA and RPL23aB. [file 12870_2020_2672_MOESM1_ESM.docx]

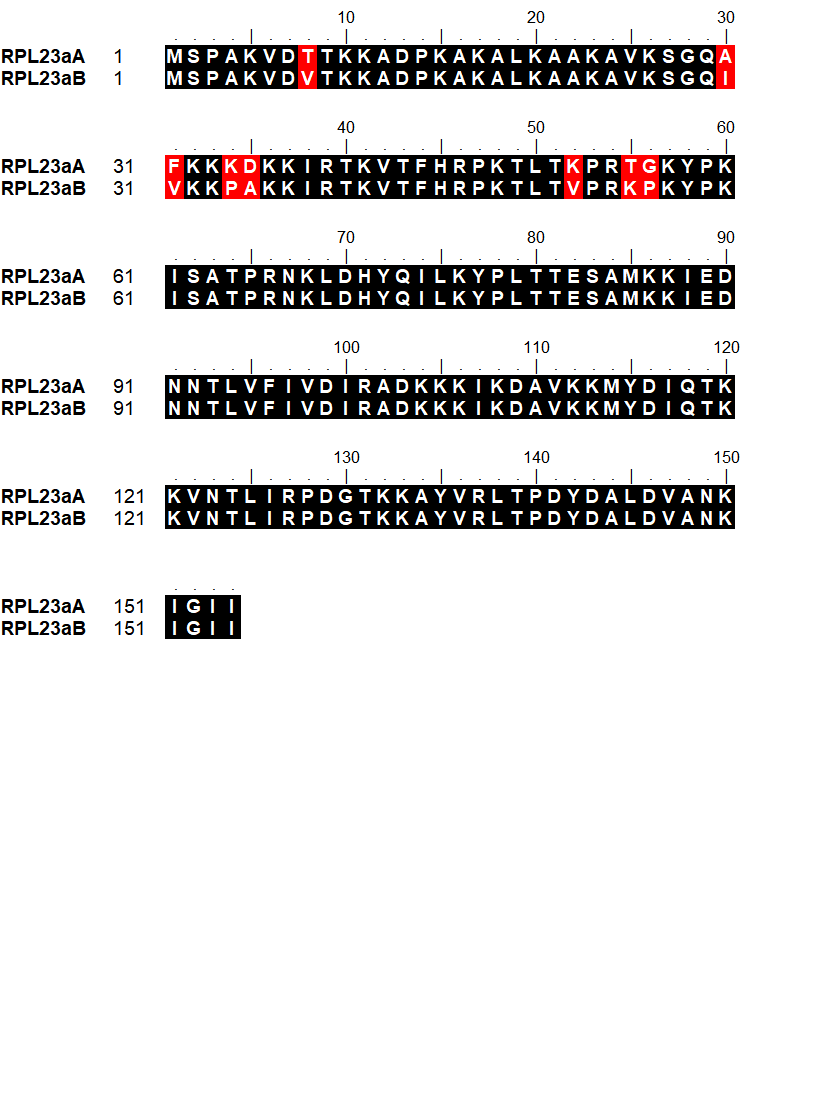


**Figure S1. Amino acid sequence alignment between RPL23aA and RPL23aB.** RPL23aA and RPL23aB are 95% identical in amino acid sequences. The black background indicates identical amino acids. The different amino acids are in red highlight.
