# Supplementary material for: Arabidopsis paralogous genes RPL23aA and RPL23aB encode functionally equivalent proteins
Source: BMC Plant Biol. 2020 Oct 8;20:463. doi: 10.1186/s12870-020-02672-1 (PMC7545930; doi:10.1186/s12870-020-02672-1)
Supplement: Supplementary file 2 — Additional file 2: Figure S2. Genotyping of rpl23aa and rpl23ab. [file 12870_2020_2672_MOESM2_ESM.docx]

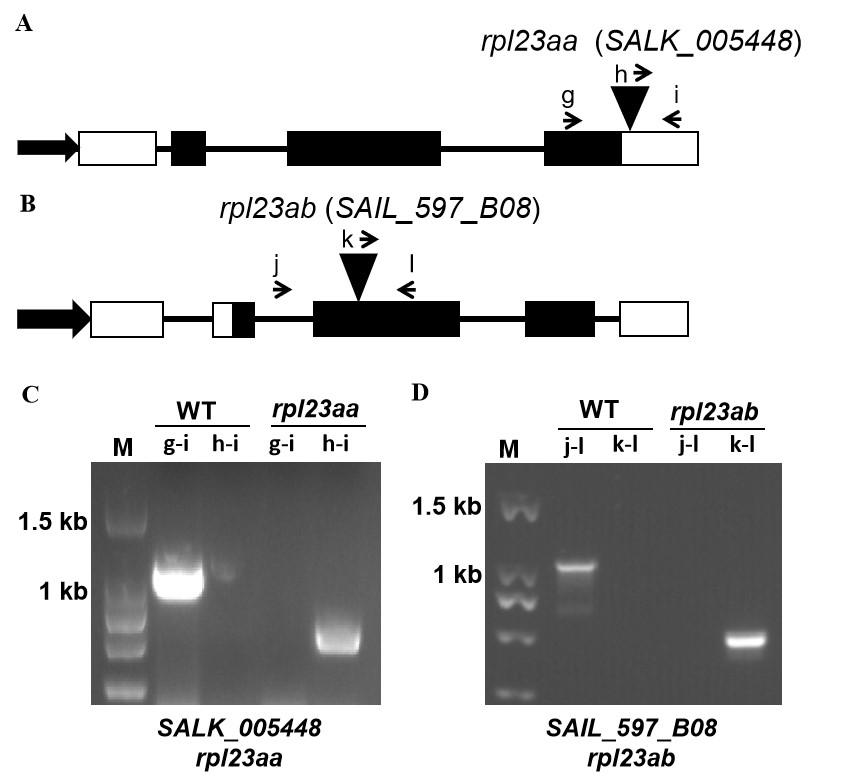


**Figure S2. Genotyping of *rpl23aa* and *rpl23ab.*** (**A**), (**B**) Diagrams of *RPL23aA* and *RPL23aB* genes. The triangles indicate the positions of the T-DNA insertion in the two mutants. Black boxes and lines between black boxes represent exons and introns, respectively. The white rectangles correspond to the 5’ and 3’ untranslated regions. The thick arrows indicate promoters and the small arrows represent primers used in PCR reactions in (**C**) and (**D**). (**C**) *RPL23aA* and (**D**) *RPL23aB* DNA were amplified by the specified primer pairs from wild type and mutants. The size of PCR products: g-i 1.2 kb, h-i 750 bp, j-l 1.2 kb, k-l 750 bp. The full-length gel of (**C**) was shown in Figure S10 (Additional file 10) and the original image of (**D**) was shown in Figure S11 (Additional file 11) .
