# Supplementary material for: Arabidopsis paralogous genes RPL23aA and RPL23aB encode functionally equivalent proteins
Source: BMC Plant Biol. 2020 Oct 8;20:463. doi: 10.1186/s12870-020-02672-1 (PMC7545930; doi:10.1186/s12870-020-02672-1)
Supplement: Supplementary file 4 — Additional file 4: Figure S4. Lengths of mature siliques and numbers of ovules in mature siliques. [file 12870_2020_2672_MOESM4_ESM.docx]

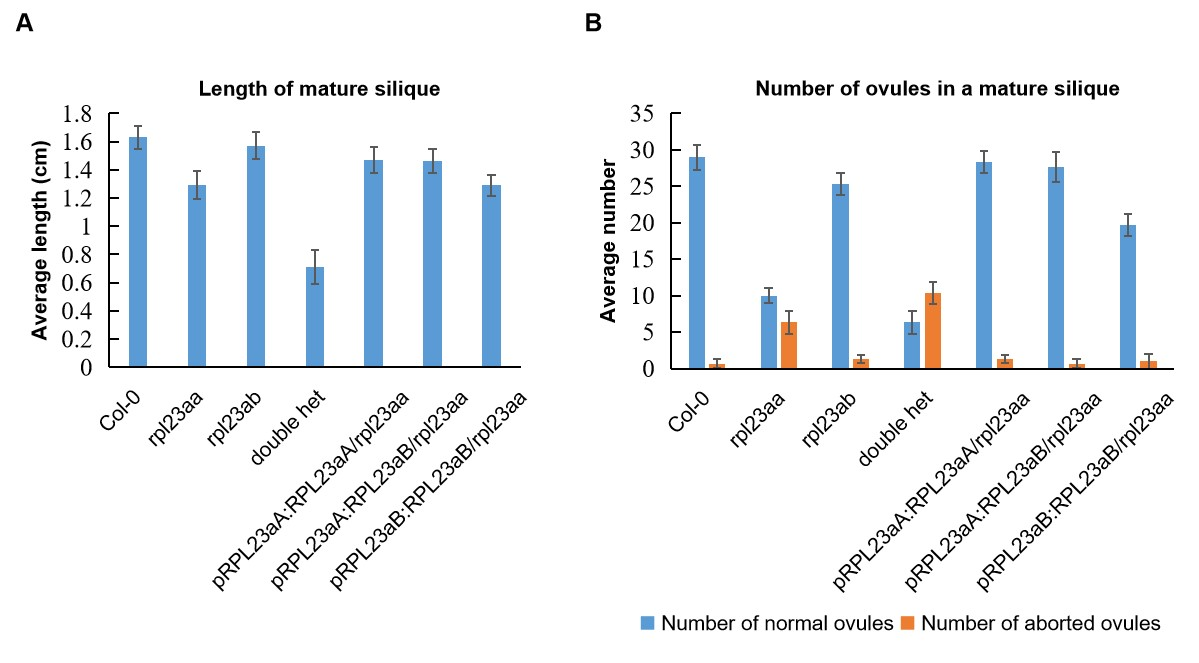


**Figure S4. Statistic of length of mature silique and number of ovules in a mature silique.** (A) average length of mature siliques from Col-0, *rpl23aa*, *rpl23ab*, double heterozygote, *pRPL23aA::RPL23aA/rpl23aa*, *pRPL23aA::RPL23aB/rpl23aa*, and *pRPL23aB::RPL23aB/rpl23aa* plant. (B) average number of normal and aborted ovules.
