# Supplementary material for: Arabidopsis paralogous genes RPL23aA and RPL23aB encode functionally equivalent proteins
Source: BMC Plant Biol. 2020 Oct 8;20:463. doi: 10.1186/s12870-020-02672-1 (PMC7545930; doi:10.1186/s12870-020-02672-1)
Supplement: Supplementary file 5 — Additional file 5: Figure S5. Images of pRPL23aB:RPL23aB rpl23aa plants. [file 12870_2020_2672_MOESM5_ESM.docx]

**
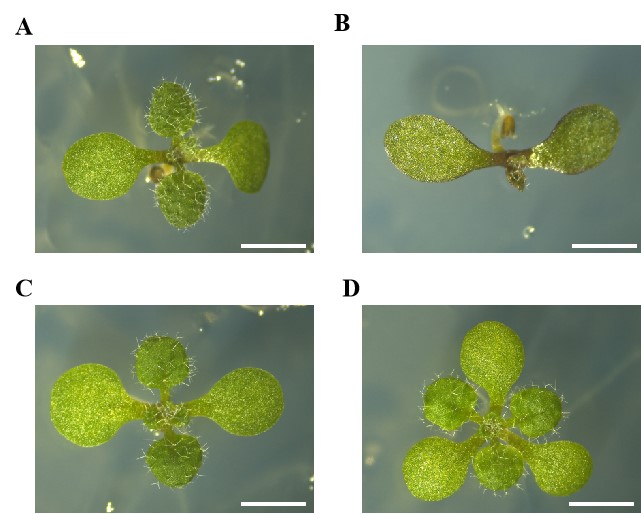
**

**Figure S5. Images of *pRPL23aB:RPL23aB* *rpl23aa* plants.** 9-day-old plants of (**A**) Col-0, (**B**) *rpl23aa*, (**C**) a *pRPL23aB::RPL23aB/rpl23aa* plant with two cotyledons*,* (**D**) a *pRPL23aB::RPL23aB/rpl23aa* plant with three cotyledons. Size bar, 5 mm.
