# Supplementary material for: Arabidopsis paralogous genes RPL23aA and RPL23aB encode functionally equivalent proteins
Source: BMC Plant Biol. 2020 Oct 8;20:463. doi: 10.1186/s12870-020-02672-1 (PMC7545930; doi:10.1186/s12870-020-02672-1)
Supplement: Supplementary file 6 — Additional file 6: Figure S6. Transcript profiles of RPL23aA and RPL23aB in different organs. [file 12870_2020_2672_MOESM6_ESM.docx]

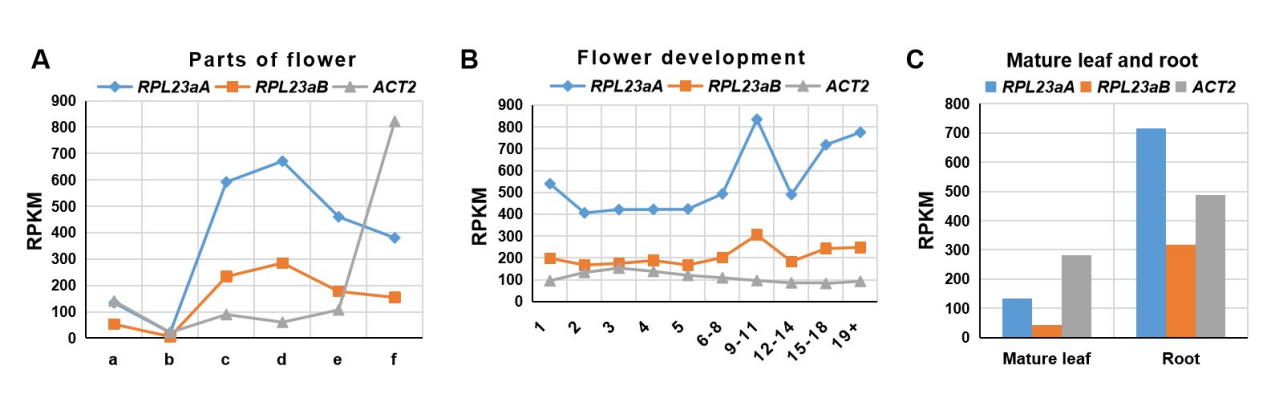


**Figure S6. Transcript profiles of *RPL23aA* and *RPL23aB* in different organs.** Y axis: the average RPKM (Reads Per Kilobase per Million mapped reads) value of two biological replicates. (**A**) parts of flower. (**B**) different flowers in the inflorescence, with the first flower at the moment of anthesis. a, sepals; b, anthers; c, carpels; d, ovules from carpels; e, stigmatic tissue; f, stamen filaments of mature flowers. (C) mature leaf and root.
