# Supplementary material for: Arabidopsis paralogous genes RPL23aA and RPL23aB encode functionally equivalent proteins
Source: BMC Plant Biol. 2020 Oct 8;20:463. doi: 10.1186/s12870-020-02672-1 (PMC7545930; doi:10.1186/s12870-020-02672-1)

**Figure S7. Polysome profiles of Col-0 (black), *rpl23aa* (red), *rpl23ab* (green), *pRPL23aA:RPL23aA/rpl23aa* (yellow), *pRPL23aA:RPL23aB/rpl23aa* (blue), and *pRPL23aB:RPL23aB/rpl23aa* (purple)**. Polysomes were extracted from 14-day-old seedlings. The absorbance peaks at 254 nm representing the 40S small subunits, 60S large subunits, 80S monosomes, and polysomes are labeled above the peaks.
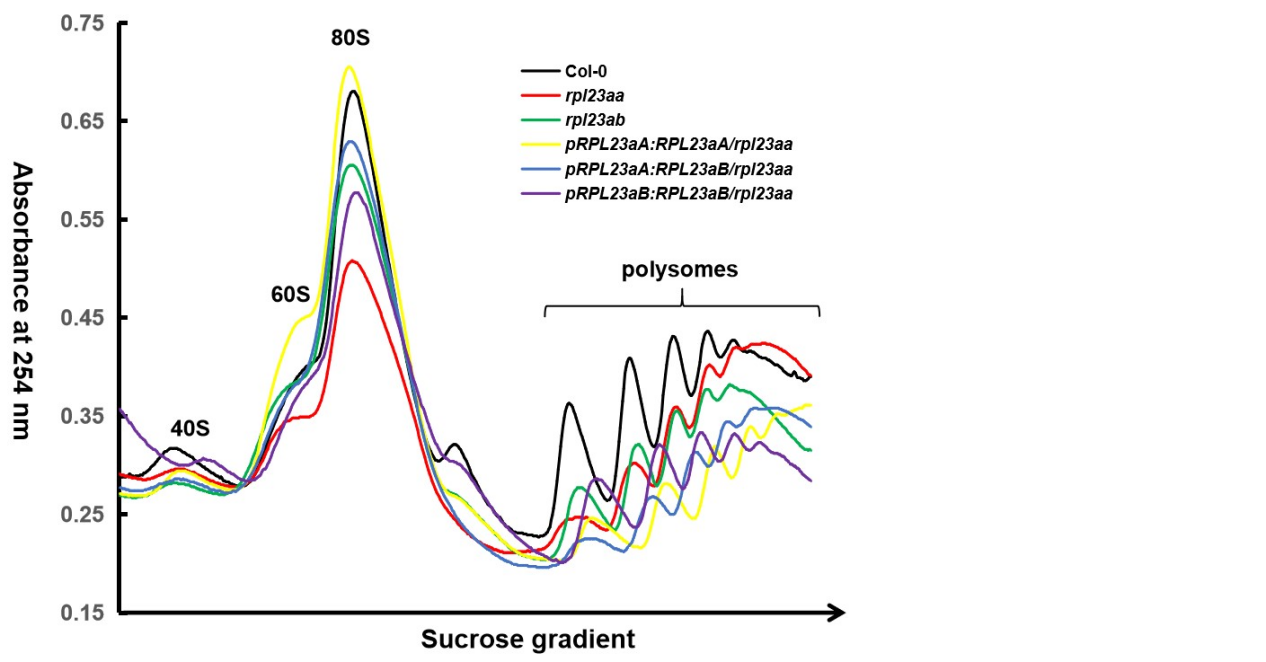

Supplement: Supplementary file 7 — Additional file 7: Figure S7. Polysome profiles of Col-0 (black), rpl23aa (red), rpl23ab (green), pRPL23aA:RPL23aA/rpl23aa (yellow), pRPL23aA:RPL23aB/rpl23aa (blue), and pRPL23aB:RPL23aB/rpl23aa (purple). [file 12870_2020_2672_MOESM7_ESM.docx]
