# Supplementary material for: Arabidopsis paralogous genes RPL23aA and RPL23aB encode functionally equivalent proteins
Source: BMC Plant Biol. 2020 Oct 8;20:463. doi: 10.1186/s12870-020-02672-1 (PMC7545930; doi:10.1186/s12870-020-02672-1)
Supplement: Supplementary file 8 — Additional file 8: Figure S8. Full-length gel of Fig. 1c. [file 12870_2020_2672_MOESM8_ESM.docx]

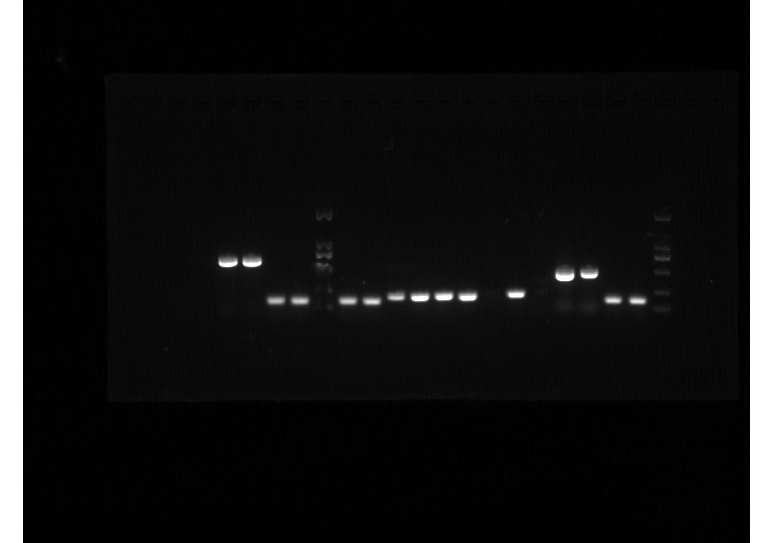


*UBQ5*

a-b

c-d

Col-0

rl23aa

Col-0

rl23aa

Col-0

rl23aa

**Figure S8. Full-length gel of Figure 1C.**

Figure 1C was cropped from Figure S8 as indicated.
