# Supplementary material for: Arabidopsis paralogous genes RPL23aA and RPL23aB encode functionally equivalent proteins
Source: BMC Plant Biol. 2020 Oct 8;20:463. doi: 10.1186/s12870-020-02672-1 (PMC7545930; doi:10.1186/s12870-020-02672-1)
Supplement: Supplementary file 9 — Additional file 9: Figure S9. Full-length gel of Fig. 1d. [file 12870_2020_2672_MOESM9_ESM.docx]

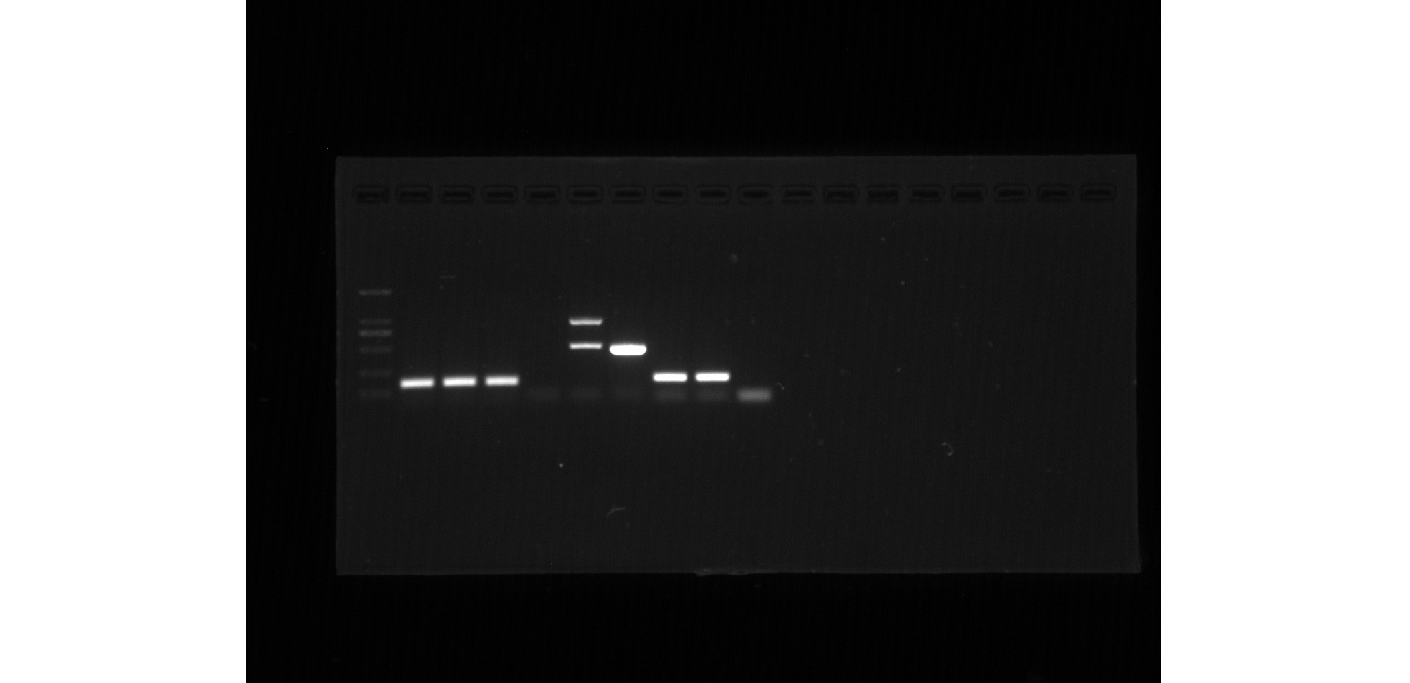


*UBQ5*

*e-f*

rl23aa

rl23ab

Col-0

rl23ab

rl23aa

Col-0

**Figure S9. Full-length gel of figure 1D.**

Figure 1D was cropped from Figure S9 as indicated.
