# Supplementary material for: Arabidopsis paralogous genes RPL23aA and RPL23aB encode functionally equivalent proteins
Source: BMC Plant Biol. 2020 Oct 8;20:463. doi: 10.1186/s12870-020-02672-1 (PMC7545930; doi:10.1186/s12870-020-02672-1)
Supplement: Supplementary file 10 — Additional file 10: Figure S10. Full-length gel of Figure S2C. [file 12870_2020_2672_MOESM10_ESM.docx]

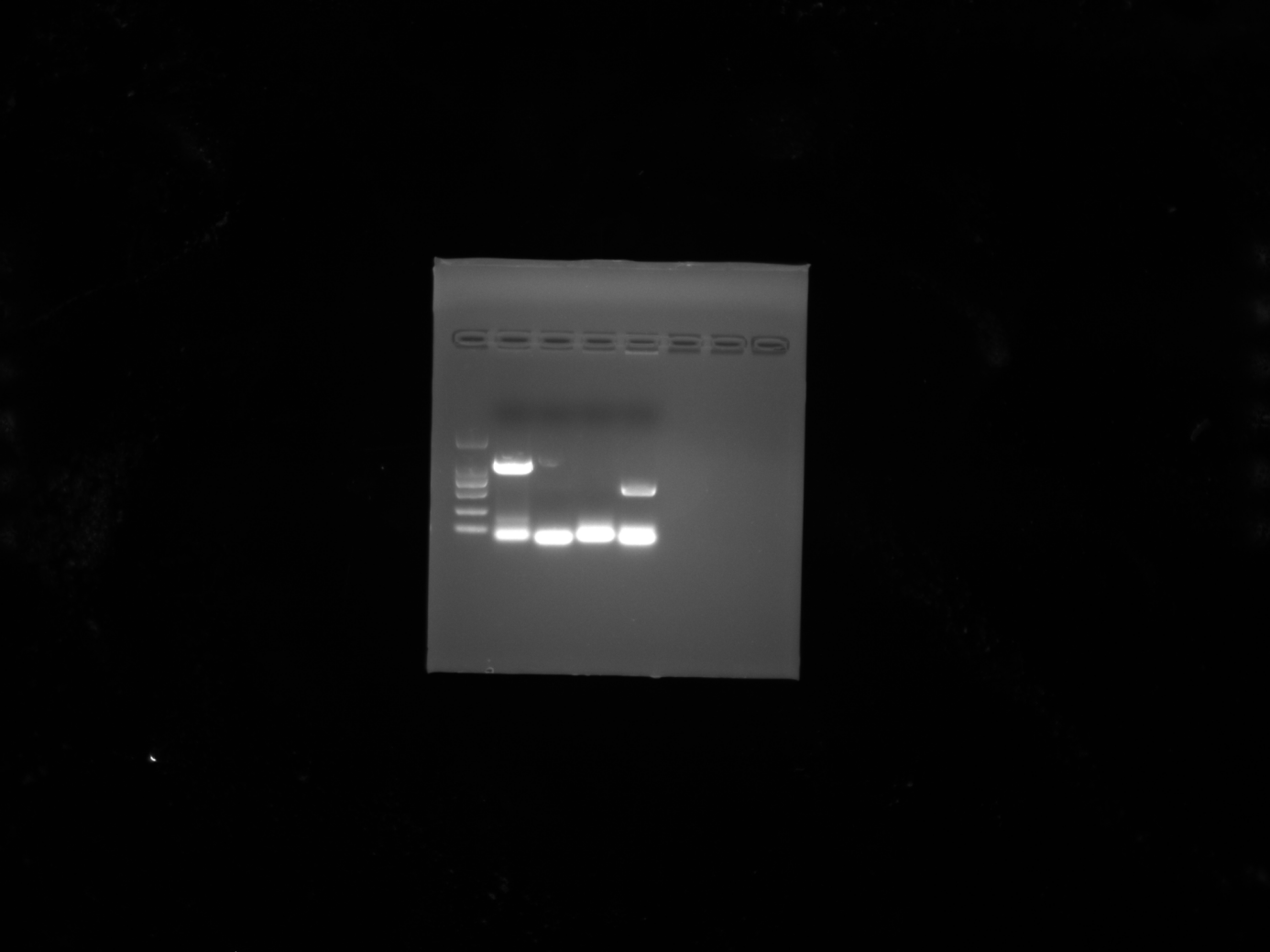


**1.5 kb**

**1 kb**

**M**

**WT**

**h-i**

**g-i**

***rpl23aa***

**g-i**

**h-i**

**Figure S10. Full-length gel of figure S2C.**

Figure S2C was cropped from Figure S10 as indicated.
