# Supplementary material for: Arabidopsis paralogous genes RPL23aA and RPL23aB encode functionally equivalent proteins
Source: BMC Plant Biol. 2020 Oct 8;20:463. doi: 10.1186/s12870-020-02672-1 (PMC7545930; doi:10.1186/s12870-020-02672-1)
Supplement: Supplementary file 11 — Additional file 11: Figure S11. Full-length gel of Figure S2D. [file 12870_2020_2672_MOESM11_ESM.docx]

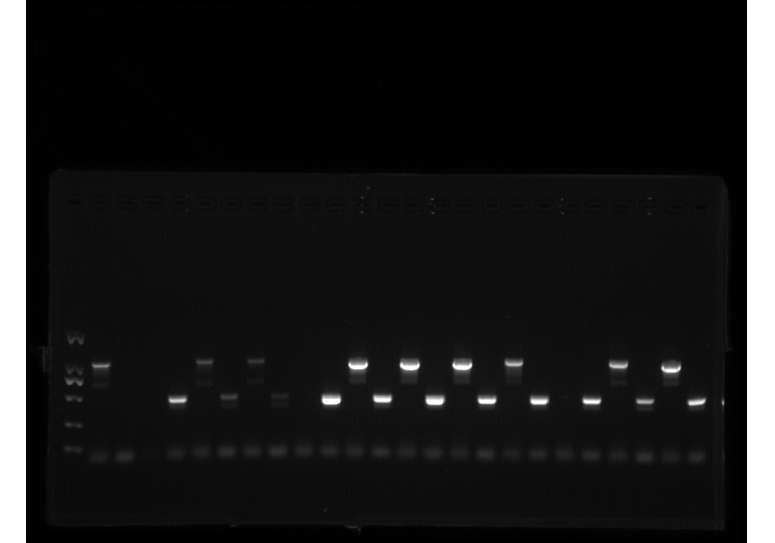


**1.5 kb**

**1 kb**

**M**

**WT**

**j-l**

**k-l**

***rpl23ab***

**j-l**

**k-l**

**Figure S11. Full-length gel of figure S2D.**

Figure S2D was cropped from Figure S11 as indicated.
