# Supplementary material for: Arabidopsis paralogous genes RPL23aA and RPL23aB encode functionally equivalent proteins
Source: BMC Plant Biol. 2020 Oct 8;20:463. doi: 10.1186/s12870-020-02672-1 (PMC7545930; doi:10.1186/s12870-020-02672-1)
Supplement: Supplementary file 12 — Additional file 12: Table S1. Primers used in this work. [file 12870_2020_2672_MOESM12_ESM.docx]

**Table S1. Primers used in this work.**

| **Primer ID** | **Primer sequence** | **Use** |
| --- | --- | --- |
| SALK_BP (Primer h) | ATTTTGCCGATTTCGGAAC | *rpl23aa* genotyping |
| SALK_005448_LP (Primer g) | TGCTGAACCCGCTAGTATCTG | *rpl23aa* genotyping |
| SALK_005448_RP (Primer i) | GTGAGGAGACTGCCATAGTGG | *rpl23aa* genotyping |
| SAIL_BP (Primer k) | GCGTGGACCGCTTGCTGCAAC | *rpl23ab* genotyping |
| SAIL_597_B08_LP (Primer j) | TGATTAGATTGGGCTCCACAG | *rpl23ab* genotyping |
| SAIL_597_B08_RP (Primer l) | GTCTCCAGCTAAAGGTACGCC | *rpl23ab* genotyping |
| 23aA-RT -295F (Primer a) | AAGTACCCAAAAATCAGCGCTAC | RT-PCR |
| 23aA-RT-510R (Primer b) | AGGCCTGATGAGTGTGTTCA | RT-PCR |
| 23aA-RT -402F (Primer c) | CACTCTTGTTTTCATTGTTG | RT-PCR |
| 23aA-RT-607R (Primer d) | GCAGAGATAGATAGACTTAGATGA | RT-PCR |
| 23aB-RT-F (Primer e) | CGTGAAAAGAATCTATCTTGAGCA | RT-PCR |
| 23aB-RT-R (Primer f) | AGGCTTTCTAGGAACGGTCAATG | RT-PCR |
| UBQ5 F | GGTGCTAAGAAGAGGAAGAA | RT-PCR |
| UBQ5 R | CTCCTTCTTTCTGGTAAACGT | RT-PCR |
| 23aA promoter F | CGGGGTACCTATTTGTTGACATTACTTCCA | Promoter-GUS |
| 23aA promoter R | CGCGGATCCGGCTTGAAATGATTCTTCAC | Promoter-GUS |
| 23aB promoter F | CGGGGTACCGGCTTCACCTTCTCTTGCTG | Promoter-GUS |
| 23aB promoter R | CGCGGATCCTGCTCAAGATAGATTCTTTT | Promoter-GUS |
| 23aA genomic DNA F | CGGGGTACCAGCCACTAGTGACAGAAGC | Complementation |
| 23aA genomic DNA R | CGCGGATCCGATGATGCCGATCTTGTTAG | Complementation |
| 23aB genomic DNA F | CGGGGTACCTCAGCTCAGGTGAGAATTCT | Complementation |
| 23aB genomic DNA R | CGCGGATCCGATGATCCCGATTTTGTTAG | Complementation |
